# Duck (*Anas platyrhynchos*) linkage mapping by AFLP fingerprinting

**DOI:** 10.1186/1297-9686-41-28

**Published:** 2009-03-17

**Authors:** Chang-Wen Huang, Yu-Shin Cheng, Roger Rouvier, Kuo-Tai Yang, Chean-Ping Wu, Hsiu-Lin Huang, Mu-Chiou Huang

**Affiliations:** 1 Department of Animal Science, National Chung Hsing University, 250 Kuo-Kung Road, Taichung 402, Taiwan; 2 Institute of Cellular and Organism Biology, Academia Sinica, 128 Section 2, Academia Road, Nankang, Taipei 115, Taiwan; 3 Livestock Research Institute, Council of Agriculture, Hsin-Hua, Tainan 712, Taiwan; 4 Institut National de la Recherche Agronomique, Station d'Amélioration Génétique des Animaux, Centre de Recherches de Toulouse, BP52627, F31326 Castanet-Tolosan Cedex, France; 5 Institute of Biomedical Sciences, Academia Sinica, 128 Section 2, Academia Road, Nankang, Taipei 115, Taiwan; 6 Department of Animal Science, National Chiayi University, 300 Syuefu Road, Chiayi 600, Taiwan

## Abstract

Amplified fragment length polymorphism (AFLP) with multicolored fluorescent molecular markers was used to analyze duck (*Anas platyrhynchos*) genomic DNA and to construct the first AFLP genetic linkage map. These markers were developed and genotyped in 766 F2 individuals from six families from a cross between two different selected duck lines, brown Tsaiya and Pekin. Two hundred and ninety-six polymorphic bands (64% of all bands) were detected using 18 pairs of fluorescent *Taq*I/*Eco*RI primer combinations. Each primer set produced a range of 7 to 29 fragments in the reactions, and generated on average 16.4 polymorphic bands. The AFLP linkage map included 260 co-dominant markers distributed in 32 linkage groups. Twenty-one co-dominant markers were not linked with any other marker. Each linkage group contained three to 63 molecular markers and their size ranged between 19.0 cM and 171.9 cM. This AFLP linkage map provides important information for establishing a duck chromosome map, for mapping quantitative trait loci (QTL mapping) and for breeding applications.

## Introduction

Amplified fragment length polymorphism (AFLP) is an application of the DNA fingerprinting technique proposed by Vos *et al*. [1], which is a clever combination of two older methods, restriction fragment length polymorphism (RFLP) [2] and random amplified polymorphic DNA (RAPD) [3-5], generating a large number of genetic markers from any genomic DNA [6]. AFLP markers are inherited in a Mendelian fashion and can be detected as co-dominant markers [7]. Since Ajmone-Marsan *et al*. [8], several studies have shown that AFLP markers follow Mendelian inheritance rules and that the technique is highly reproducible, powerful and efficient [9]. Thus AFLP analysis is a useful tool to generate linkage maps [10].

Linkage maps using AFLP, microsatellite or SNP markers have been established and applied extensively to linkage studies or quantitative trait locus (QTL) mapping in animals such as rats [11], rabbits [12], goats [13], sheep [14], cattle [15], chickens [16-20], turkeys [21], quails [22,23], and fish [24,25]. They have also been much used for genome mapping, studies on disease resistance and drug tolerance in economic crops and other experimental plants such as sorghum [26], *Arabidopsis thaliana *[27], rice [28], corn [29], barley [30] and wheat [31].

Ducks are appreciated for meat and eggs. Research on duck genetics and breeding has been developed only in recent years [32]. For detecting and mapping QTL, the construction of a genetic linkage map is a prerequisite and in duck genetic map data are very limited. Huang *et al*. [33] have reported a preliminary genetic linkage map in an inbred Pekin ducks resource population using microsatellite markers. The advantage of AFLP is that a large number of markers can be generated with a smaller number of primer pairs than required when using microsatellites. This is especially true when working in a species for which only few microsatellite markers are available. A large number of microsatellite markers may be obtained if enough time and financial support are available. In this study, we have chosen the AFLP technique to develop a duck genetic map. We have used the *Taq*I/*Eco*RI restriction enzyme combination and selective PCR primers to generate molecular genetic markers and to establish a duck genetic linkage map from a resource population originating from a cross between two outbred selected lines of laying and meat type ducks. This is a first step to provide vital information to construct chromosome maps and map QTL for future applications in duck breeding.

## Methods

### Animals and blood collection

All ducks tested in the study originate from the Livestock Research Institute, Council of Agriculture (LRI-COA). In the first generation F0, each of three brown Tsaiya drakes and three Pekin drakes were mated either to two Pekin ducks or to two brown Tsaiya female ducks, respectively. Six F1 drakes originating from the six F0 sires were mated individually, according to the mating plan, with three (one case) or six (five cases) unrelated F1 dams that were daughters of one F0 drake of the same breed brown Tsaiya or Pekin. F2 birds belonging to six half-sib families were used as the mapping population. The number of birds in the resource population was as follows: six males and 12 females in the F0, six males and 33 females in the F1 and 766 males and females in the F2. A total of 766 F2 animals were genotyped. Blood samples obtained from the vein of the ducks wings were carefully mixed with anticoagulant and kept at 4°C for subsequent DNA extraction.

### Genomic DNA extraction

DNA extraction procedures were performed according to the method described by Huang *et al*. [34]. Eighty μL of each blood sample were mixed thoroughly with 1 mL of TNE buffer solution (10 mM Tris-HCl pH 8.0, 150 mM NaCl, 10 mM EDTA pH 8.0) in a 1.5 mL centrifuge tube and centrifuged at 1,500 × g (Hermle Model Z233 MK, Maryland, USA) for 5 min to wash the cells. They were then resuspended in 300 μL 10% NH_4_Cl, 75 μL proteinase K (10 mg/mL), 25 μL collagenase (3.8 IU/μL), and 200 μL 10% w/v SDS and the mixture was incubated at 42°C for 24 h, with agitation. A series of extractions was performed with a same volume of phenol, phenol/chloroform (containing 1/25 v/v isoamyl alcohol), and chloroform, respectively. Centrifugation conditions were 3,000 × g (Model SCT5B, HITACHI) for 10 min, then samples were precipitated with isopropanol. Excess isopropanol was removed using 70% ethanol. The DNA was vacuum-dried (Speed Vac^® ^SC110, Rotor RH 40-11, SAVANT) and resuspended in double distilled water. The DNA was quantified with an S2000 UV/Vis Diode-Array Spectrophotometer (WAP Co. Ltd., Cambridge, UK) to determine its absorbance and to confirm DNA purity and concentration for AFLP analysis.

### Analysis of genotypes using AFLP markers

AFLP analysis was carried out according to the procedures described by Vos *et al*. [1]. All sequences for the *Eco*RI and *Taq*I adapters and primers used in this study are shown in Table 1. Briefly, 400–500 ng of genomic DNA (50 ng/μL) was digested with 0.5 μL *Eco*RI restriction endonuclease (20 U/μL) with 1 μL of 10× *Eco*RI buffer (50 mM NaCl, 100 mM Tris-HCl, 10 mM MgCl_2_, 0.025% Triton X-100, pH 7.5) (New England BioLabs^® ^Inc., Ipswich, MA, USA) in a total volume of 10 μL. The mixture was incubated at 37°C for 4 h and then at 65°C for 10 min. Subsequently, the sample was digested with 0.5 μL *Taq*I restriction endonuclease (20 U/μL) with 1.5 μL of 10× *Taq*I buffer (100 mM NaCl, 10 mM Tris-HCl, 10 mM MgCl_2 _pH 8.4) (New England BioLabs^® ^Inc., Ipswich, MA, USA), then mixed with 0.15 μL of 100× BSA in a total volume of 15 μL and incubated at 65°C for 4 h with a last step at 80°C for 10 min. Adaptor ligation was performed by adding 1 μL of *Taq*I-adaptor (50 ng/μL), 0.1 μL of *Eco*RI-adaptor (50 ng/μL), 1 μL of T4 DNA ligase (1 U/μL) and 5 μL of 5× ligase buffer (250 mM Tris-HCl pH 7.6, 50 mM MgCl_2_, 5 mM ATP, 5 mM DTT, 25% polyethylene glycol-8000) (Invitrogen Co., Carlsbad, CA, USA). The mixture was made up to 25 μL with double-distilled water and incubated at 23°C for 12 h. DNA pre-amplification was performed in a GeneAmp^® ^PCR system 2700 thermocycler (Applied Biosystems, Singapore) with a final volume of 20 μL containing 6 μL of the DNA sample, 1 μL of *Taq*I+A primer (50 ng/μL), 1 μL of *Eco*RI+A primer (50 ng/μL), 1.6 μL of 2.5 mM dNTPs, 0.25 μL of DyNAzyme™ DNA polymerase (2 U/μL, F-501L, Finnzymes Oy, Espoo, Finland), and 2 μL of 10× PCR buffer (100 mM Tris-HCl pH 8.8, 15 mM MgCl_2_, 500 mM KCl, 1% Triton X-100). The following PCR conditions were used: a denaturing step for 5 min at 94°C, 20 cycles at 94°C for 30 s, 56°C for 1 min and 72°C for 1 min and a final extension step at 72°C for 5 min. A second PCR reaction was performed in a final volume of 5 μL containing 0.5 μL of the pre-amplification PCR products, 1 μL of *Taq*I+A*NN *selective primer (50 ng/μL), 1 μL fluorescent dye-labeled *Eco*RI+A*NN *selective primer (50 ng/μL) with either VIC (green), NED (yellow), PET (red) or FAM (blue) 0.3 μL of 2.5 mM dNTPs, 0.25 μL of DyNazyme™ DNA polymerase (2 U/μL), and 0.5 μL of 10× buffer (10 mM Tris-HCl pH 8.3, 1.5 mM MgCl_2_, 50 mM KCl). Conditions for the selective amplification PCR are shown in Table 2.

**Table 1:** Sequences of adapters and primers used in the AFLP detection

	**Name**	**Sequence**
Adapter *Eco*RI		
	*Eco *Top Strand	5-CTCGTAGACTGCGTACC
	*Eco *Bottom Strand	5-AATTGGTACGCAGTCTAC
Adapter *Taq*I		
	*Taq *Top Strand	5-GACGATGAGTCCTGAC
	*Taq *Bottom Strand	5-CGGTCAGGACTCAT
Primer *Eco*RI		
	*Eco*R+A	5-GAC TGC GTA CCG TAC CA
E1	VIC-*Eco*R+AAA	5-GAC TGC GTA CCG TAC CAA A
E2	NED-*Eco*R+AAC	5-GAC TGC GTA CCG TAC CAA C
E3	PET-*Eco*R+AAG	5-GAC TGC GTA CCG TAC CAA G
E4	FAM-*Eco*R+ACA	5-GAC TGC GTA CCG TAC CAC A
E5	VIC-*Eco*R+AC	5-GAC TGC GTA CCG TAC CAC
E6	FAM-*Eco*R+AG	5-GAC TGC GTA CCG TAC CAG
Primer *Taq*I		
	*Taq*+A	5-GAT GAG TCC TGA CCG AA
T1	*Taq*+AAC	5-GAT GAG TCC TGA CCG AAA C
T2	*Taq*+AAG	5-GAT GAG TCC TGA CCG AAA G
T3	*Taq*+AAT	5-GAT GAG TCC TGA CCG AAA T
T4	*Taq*+ACA	5-GAT GAG TCC TGA CCG AAC A
T5	*Taq*+AC	5-GAT GAG TCC TGA CCG AAC
T6	*Taq*+AG	5-GAT GAG TCC TGA CCG AAG

**Table 2:** Conditions of selective amplification PCR

**Hold**	**Cycle**			**Number of cycles**
94°C, 5 min	94°C, 30 s	66°C, 30 s	72°C, 1 min	2
-	94°C, 30 s	64°C, 30 s	72°C, 1 min	2
-	94°C, 30 s	62°C, 30 s	72°C, 1 min	2
-	94°C, 30 s	60°C, 30 s	72°C, 1 min	2
-	94°C, 30 s	58°C, 30 s	72°C, 1 min	2
-	94°C, 30 s	56°C, 30 s	72°C, 1 min	25
-	-	-	72°C, 5 min	1
4°C, forever	-	-	-	1

Equal volumes of each of the four PCR products with different color fluorescent markers (either VIC, NED, PET or FAM) were combined, diluted and mixed with double-distilled water and mixed. Then, 1 μL of the diluted PCR product mixture was added to 0.2 μL of GeneScan-500 LIZ internal lane size standard (Applied Biosystems, Foster City, CA, USA) and 10.8 μL of deionized formamide, denatured for 3 min at 94°C and immediately after placed on ice for 5 min. Capillary electrophoresis was performed on an ABI PRISM^® ^3100 Avant Genetic Analyzer using the GS STR POP-6 F module column (Applied Biosystems, Foster City, CA, USA). Fluorescent peak signals for each primer combination were collected with the ABI PRISM^® ^3100 Genetic Analyzer Data Collection 1.1 (Applied Biosystems, Foster City, CA, USA). The resulting genotyping data were scanned and analyzed with the software ABI PRISM™ GeneScan 3.7 and Genotyper 3.7 software package (Applied Biosystems, Foster City, CA, USA), which displayed the AFLP fingerprints and quantified the polymorphic peaks. AFLP markers were named according to the serial number based on the extension sequence of *Taq*I and *Eco*RI primer combination (Table 3) and to the size of the fragment in base pairs. Polymorphic markers from duck individuals belonging to the same family were scored according to the different heights and distributions of peak signals using the Genotyper software.

**Table 3:** Number of detected polymorphisms per primer pair 3' end extensions of *Eco*RI and *Taq*I primers are shown; *Eco*RI primers are fluorescently labeled

**Primer extensions^1^**		**Nb of peaks**	**Polymorphic markers**		**Mapped marker**		
			
*Taq*I	*Eco*RI, labeled		No.	%	No.	%	Size range of peaks (bp)
AAC	AAA, VIC	33	20	61	18	90	61–399
AAC	AAC, NED	28	19	68	13	68	60–260
AAC	AAG, PET	15	9	60	8	89	84–282
AAC	ACA, FAM	34	24	71	21	88	91–467
AAG	AAA, VIC	36	22	61	18	82	41–261
AAG	AAC, NED	14	9	64	8	89	61–205
AAG	AAG, PET	17	11	65	11	100	45–195
AAG	ACA, FAM	21	13	62	13	100	46–349
AAT	AAA, VIC	41	25	61	21	84	44–325
AAT	AAC, NED	12	8	67	7	88	52–216
AAT	AAG, PET	16	9	56	7	78	108–282
AAT	ACA, FAM	29	18	62	17	94	91–-239
ACA	AAA, VIC	27	20	74	19	95	39–354
ACA	AAC, NED	23	14	61	12	86	39–284
ACA	AAG, PET	19	13	68	10	77	41–233
ACA	ACA, FAM	13	7	54	7	100	81–283
AC	AC, VIC	42	26	62	23	88	46–349
AG	AG, FAM	45	29	64	27	93	56–382
		465	296	64	260	88	

^1 ^Sequence of the two or three selective nucleotides at the 3' end of the AFLP primer

### Construction of linkage maps

Each polymorphic marker was analyzed by Chi-square tests. Markers heterozygous in both F1 parents and significantly (*P *= 0.05) fitting a 1:2:1 ratio (Mendelian inheritance) with the ratio of the numbers of individual genotypes A, H and B, were counted. Linkage analysis was performed by CarteBlanche software (Keygene, Wageningen, Netherlands) following the instructions of the manufacturer. Briefly, each F2 genotype data from every family was imported. Linkage groups were constructed by the 'linkage phase establishment' function, calculating the recombination frequency (*θ*) between pairs of markers and the decimal logarithm of the odds ratio score (LOD score). Significant linkage was defined by a LOD score ≥ 3.0. Map distances were calculated according to the Kosambi mapping function. The linkage maps were drawn by MapChart 2.2 [35] and denominated in accordance to the calculated length orders of linkage groups.

## Results

### Polymorphisms of fluorescent markers

The number and the size range of the detected AFLP polymorphisms are shown in Table 3. Two hundred and ninety-six polymorphic markers (64% of all peaks) were produced. Each primer pair produced between seven and 29 polymorphic markers (16.4 markers on average). This indicated that multicolor fluorescence detection with AFLP markers is a high throughput, timesaving and easily analyzed DNA fingerprinting technique. It can be applied to investigate genetic linkage and polymorphism in a population.

### Linkage mapping

**Figure 1:**
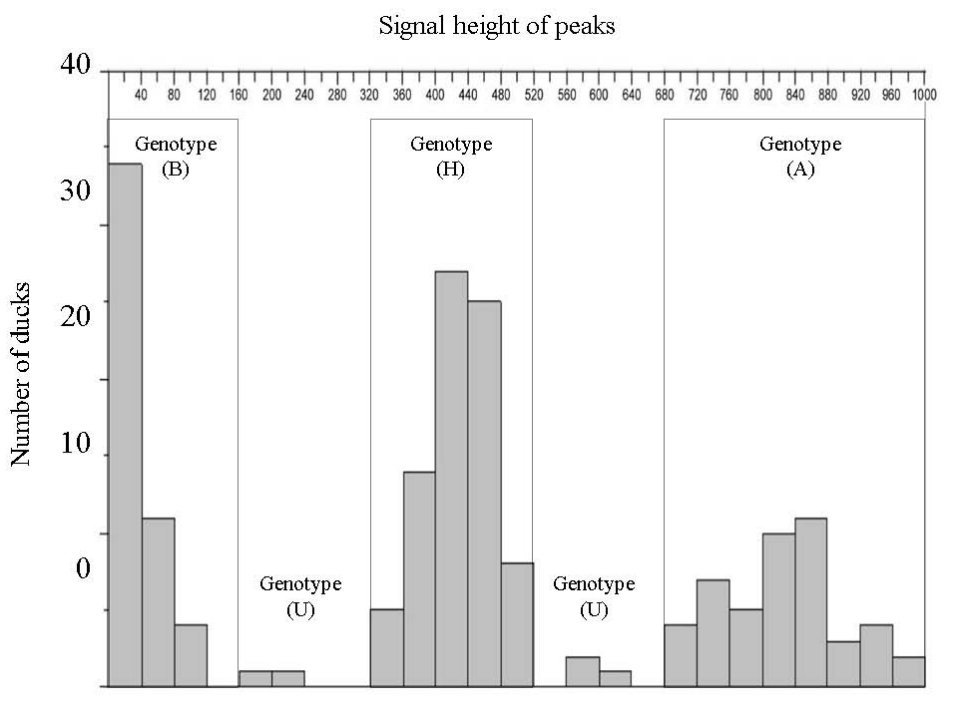
**Histogram created by ABI PRISM™ Genotyper 3.7 of signal heights from an AFLP marker in 179 F2 ducks from a single half-sib family**. Three categories are manually defined, displaying signals characterized as genotype (B) when the marker is homozygous absent, genotype (H) when the marker is heterozygous, and genotype (A) when the marker is homozygous present. Signals outside the categories are characterized as genotype (U).

**Figure 2:**
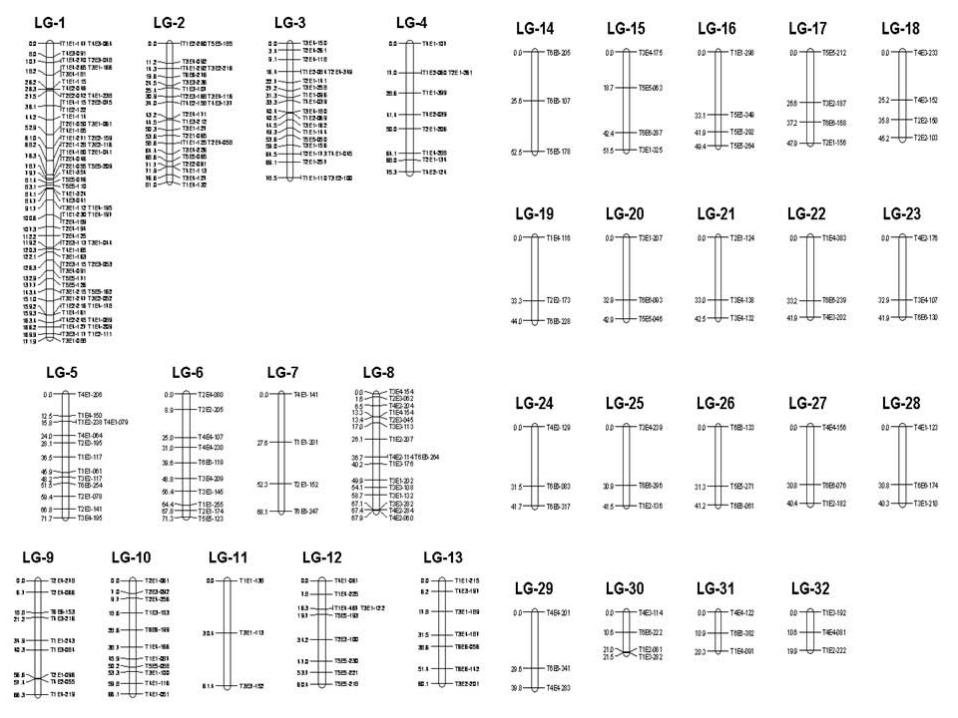
**AFLP genetic linkage map of the ducks**. Two hundred and sixty of the markers were assigned to 32 linkage groups in six families by CarteBlanche linkage software. Map distances (centimorgan, cM) were indicated to the left of the maps and calculated using the Kosambi mapping function. The names of the markers are indicated to the right of the maps.

Histograms, created by ABI PRISM™ Genotyper 3.7 of signal heights from an AFLP marker, are shown in Figure 1 and can be classified into three genotypes: homozygous present (A), heterozygous (H) and homozygous absent (B). Genotype data that were missed or could not be scored are indicated as genotype (U). After polymorphism analysis and χ^2 ^tests, 281 AFLP markers obtained from the genomic DNA of six duck families could be used for linkage analysis. Phases of all the linkage group markers were established by the 'linkage phase establishment' function in the CarteBlanche software (Keygene, Wageningen, Netherlands). Calculating recombination frequencies (*θ*), LOD scores and map distances for markers in each linkage group provided an optimum order of markers. Then, linkage maps were constructed using MapChart 2.2 [35] and they were denominated according to the calculated length orders of the linkage groups. Figure 2 shows the linkage group maps comprising 260 markers placed in 32 linkage groups. Twenty-one markers were not linked with any other marker. The number of markers in each linkage group ranged between three and 63 with 11 major groups containing 7 to 63 markers and 21 minor groups containing three to four markers. One hundred and fifty-seven of the mapped markers (60%) originated from seven linkage groups containing 10 to 63 markers. The lengths of the linkage groups varied between 19.9 and 171.9 cM. The total length of the map was 1,766 cM, with an average interval distance of 7.75 cM between two consecutive markers, the spacing between adjacent markers ranging from 0.0 cM to 33.3 cM. The results of the marker density analysis showed that the linkage group LG-1 had the highest density with 63 markers for 171 cM, whilst the LG-11 linkage group had the lowest density with three markers for 61.4 cM.

## Discussion

One purpose of the resource population produced in this work was to generate individuals with a maximum of heterozygous markers in its F1 generation. This resource population originated from a cross between two genetically different lines: a laying brown Tsaiya line selected for long duration of fertility [36,37] and a Pekin duck line selected as grand parent to produce mule ducks for roasting. Six F1 drakes from the six F0 sires were each mated with three (one case) or six (five cases) unrelated F1 dams, which were daughters of one F0 drake of the same breed brown Tsaiya or Pekin. Using AFLP markers to screen genotypes on every F2 individual from each family, we found that 281 markers (60% of all bands) conformed to Mendelian segregation. These genotype results demonstrate that pedigree information from integrated family generations is important for scoring AFLP marker genotypes. In this duck population, we observed very little segregation distortion and genotyping errors. These results show also that AFLPs can be scored as bi-allelic co-dominant markers in ducks, increasing the information content when compared to bi-allelic dominant markers and facilitating linkage and QTL analyses.

Using primer combinations labeled with multicolor fluorescent dyes and a fragment scanning system from ABI PRISM^® ^3100 Avant Genetic Analyzer, it will be possible to greatly increase the quantity and density of markers in a linkage group to build more detailed and better integrated genetic linkage maps. Due to the GC rich and gene-dense nature of bird microchromosomes [38,23], double digestion with *Eco*RI and *Taq*I restriction enzymes was performed. The sequences of adapters and primers (Table 1) and the conditions of selective amplification PCR (Table 2) were designed and adapted according to the method described by Herbergs *et al*. [19]. The average number of polymorphic fragments generated by each primer pair was 8.5 [19], 10.5 [20] in chickens and 18 in quails [23]. Our results indicate that in duck the average number of fragments is 16.4. This discrepancy may be due to species differences and to differences in the selection of primer combinations. The present results demonstrate that AFLP can produce a large amount of polymorphic markers in duck genomic DNA (Table 3). Therefore, AFLP markers are useful for linkage analysis in ducks.

For a given number of informative meiosis, the higher the LOD score, the closer the distance between two markers, which means there is a high probability that the two markers are located in the same linkage group. The map is relatively dense with an average interval distance between adjacent markers of 7.75 cM. The large number of chromosomes (2*n *= 80) and especially the presence of microchromosomes [39], make it difficult to build an exhaustive map and thus the number of linkage groups is smaller than the number of chromosome pairs. However, AFLP markers are expected to provide a better coverage of microchromosomes than microsatellite markers [38,23]. Currently, the use of AFLP marker analysis to establish a genetic linkage map is mainly restricted to plant studies [6]. A recent study applied the microsatellite technique to establish a preliminary genetic linkage map in an inbred Pekin duck resource population [33]. When comparing the results with our current study (Figure 2), AFLP markers produced a higher number of linkage groups (32 *vs *19) and an increased marker density (average interval distance 7.75 cM *vs *15.04 cM). This difference is mainly caused by the use of different molecular markers, resource populations and analysis methods. However, the microsatellite map made it possible to construct in parallel a cytogenetic map, which is not possible with AFLP markers. Thus, AFLP and microsatellite markers each have their advantages and drawbacks. To date, no large and integrated duck map is available for analysis and comparison. The successful establishment of a duck linkage map using AFLP genetic markers (Figure 2) in this study provides important information to integrate the published microsatellite markers, to set up a duck chromosome map, to map QTL and to develop future breeding applications.

## Competing interests

The authors declare that they have no competing interests.

## Authors' contributions

C-WH carried out the AFLP detection, performed the construction of the map, and wrote the first draft of the manuscript. Y-SC participated in the design and supervising the study, provided the duck samples, pedigree and performance information. RR participated in the design and supervising of the study, directed the data analysis, and helped to improve the manuscript. K-TY and C-PW participated in the collection of samples, prepared the genomic DNA and helped to the AFLP detection. H-LH participated in the collection of samples, prepared the genomic DNA, and helped to interpret the data and draft the manuscript. M-CH conceived, designed and supervised the study, succeeded in finding funding and coordination, and finalized the manuscript. All authors read and approved the final manuscript.
